# Absorption of Vitamin A and Carotenoids by the Enterocyte: Focus on Transport Proteins

**DOI:** 10.3390/nu5093563

**Published:** 2013-09-12

**Authors:** Emmanuelle Reboul

**Affiliations:** 1 INRA, UMR1260, Nutrition, Obesity and Risk of Thrombosis, Marseille F-13385, France; E-Mail: Emmanuelle.Reboul@univ-amu.fr; Tel.: +33-4-91-29-41-03; Fax: +33-4-91-78-21-01; 2 INSERM, UMR1062, Marseille F-13385, France; 3 Aix-Marseille University, Marseille F-13385, France

**Keywords:** retinol, carotenes, xanthophylls, bioavailability, transporters, intestinal absorption

## Abstract

Vitamin A deficiency is a public health problem in most developing countries, especially in children and pregnant women. It is thus a priority in health policy to improve preformed vitamin A and/or provitamin A carotenoid status in these individuals. A more accurate understanding of the molecular mechanisms of intestinal vitamin A absorption is a key step in this direction. It was long thought that β-carotene (the main provitamin A carotenoid in human diet), and thus all carotenoids, were absorbed by a passive diffusion process, and that preformed vitamin A (retinol) absorption occurred via an unidentified energy-dependent transporter. The discovery of proteins able to facilitate carotenoid uptake and secretion by the enterocyte during the past decade has challenged established assumptions, and the elucidation of the mechanisms of retinol intestinal absorption is in progress. After an overview of vitamin A and carotenoid fate during gastro-duodenal digestion, our focus will be directed to the putative or identified proteins participating in the intestinal membrane and cellular transport of vitamin A and carotenoids across the enterocyte (*i.e*., Scavenger Receptors or Cellular Retinol Binding Proteins, among others). Further progress in the identification of the proteins involved in intestinal transport of vitamin A and carotenoids across the enterocyte is of major importance for optimizing their bioavailability.

## 1. Introduction

Vitamin A is essential for normal cell growth, cell differentiation, immunological functions and vision [1]. However, vitamin A deficiency is still a public health problem in more than half of all countries, especially in Africa and South-East Asia where meat intake is low, and particularly in young children and pregnant women. Therefore, it is a priority in health policy to improve preformed vitamin A and/or provitamin A carotenoid status of these population subgroups. A precise understanding of the molecular mechanisms involved in vitamin A intestinal absorption is a key step in this direction.

Vitamin A is found in animal-based foods as retinyl esters (mainly retinyl palmitate). In fruits and vegetables, it occurs as provitamin A carotenoids (mainly β-carotene, α-carotene and β-cryptoxanthin), which can be cleaved and metabolized into retinol after absorption by the intestinal cells (Table 1). Nonprovitamin A carotenoids, such as lutein and lycopene, share similar digestion/absorption pathways with provitamin A carotenoids: they will thus be included in this review when appropriate (Table 1). The fundamental mechanisms of preformed vitamin A and carotenoid absorption were first studied 40 years ago using rat everted intestinal sacs [2,3,4]. The data obtained suggested that carotenoids were absorbed by passive diffusion, while preformed A was absorbed via (a) carrier-dependent proteins. Recent studies completed over the past ten years have once again addressed these hypotheses, and have shown that the mechanisms of retinol and carotenoid absorption are actually more complex than previously thought. Although a passive diffusion may occur at pharmacological concentrations of these compounds, a protein-mediated transport is clearly involved at dietary doses.

After an overview of the fate of retinol and carotenoid in the human upper gastrointestinal lumen, we will focus on the putative or identified proteins participating in the intestinal membrane and cellular transport of vitamin A and carotenoids across the enterocyte identified until 2013.

## 2. Overview of Vitamin A and Carotenoid Fate during the Digestion Process

Fat-soluble micronutrients including vitamin A and carotenoids are assumed to follow the fate of lipids in the upper gastrointestinal tract [5], and their absorption presumably occurs in the upper half of the small intestine.

**Table 1:** Main dietary retinoids and carotenoids.

	Molecular structure	Main food sources (µg/100 g) [6,7,8,9]	Transport proteins in the enterocyte
Retinol	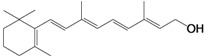	Liver: 10,800–23,500 Fatty fish: 800−1000 Butter: 700	CRBPII, possibly ABCA1
Retinyl palmitate	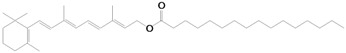		
β-carotene	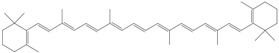	Raw carrot: 8840 Canned carrot: 5780 Cooked spinach: 5240	SR-BI, CD36
α-carotene	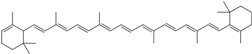	Cooked carrot: 468	
β-cryptoxanthin	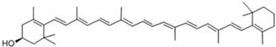	Orange juice: 880 Mandarin juice: 920	
Lutein	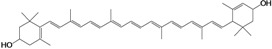	Cooked spinach: 7040 Lettuce: 2640	SR-BI, NPC1L1, possibly ABCG5
Lycopene	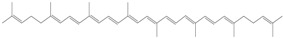	Tomato sauce: 15,920 Tomatoes: 3030 Watermelon: 4870	SR-BI

The first phase of the process of digestion/absorption is the dissolution of carotenoids [10,11] and vitamin A [12] in the fat phase of the meal. This phase is emulsified into lipid droplets in the stomach and duodenum. The size of the droplets has apparently no effect on the efficiency of the absorption of vitamin A in healthy humans, and no degradation or absorption of vitamin A has been detected at the stomach level [11,12].

It seems that only the free forms of fat-soluble vitamins and carotenoids are absorbed by the intestinal mucosa, suggesting that the esterified forms are first hydrolyzed. Studies on this topic mainly concern retinyl esters. Their hydrolysis may begin in the stomach, where gastric lipase hydrolyses about 17.5% of the triacylglycerols [13]. However, the data obtained in healthy subjects have shown that gastric lipase does not significantly hydrolyse retinyl palmitate [12]. The hydrolysis of esters of vitamin A thus occurs essentially in the duodenum. The pancreatic juice contains two main enzymes that could perform this hydrolysis: cholesterol ester hydrolase (CEH) and pancreatic lipase (LP). It has been shown that the CEH can achieve this hydrolysis *in vitro* [14,15,16,17]. However, studies in CEH-deficient mice showed that this enzyme was not significantly involved in the hydrolysis of retinyl esters *in vivo* [18,19]. Since some studies showed that the LP could hydrolyse retinyl palmitate *in vitro* [18,20,21,22] and as the CEH was not involved, it is assumed that the luminal hydrolysis of retinyl esters is achieved by the LP, together with the pancreatic lipase-related protein 2 [22]. The enzymes described above are all good candidates for the hydrolysis of esters of carotenoids. In a study on the bioavailability of lutein esters, it was suggested that CEH could allow the release of free lutein [17]. The esters that have not been hydrolyzed by LPs or CEH may be cleaved by mucosal enzymes, given that a retinyl ester hydrolase probably due to a phospholipase B [23] was identified at the brush border membrane (BBM) level of rat and human intestine [24,25]. Finally, it is conceivable that some esters are taken up intact by the intestinal cell and hydrolyzed intracellularly [8].

During the process of digestion, carotenoids and fat-soluble vitamins are incorporated with other lipids into the mixed micelles [5], presumably necessary for their absorption by the enterocyte. Mixed micelles are a mixture of phospholipids, cholesterol, lipid digestion products (such as free fatty acids, monoacylglycerols and lysophospholipids) and bile salts. Fat-soluble micronutrient transfer to mixed micelles during dietary lipid lipolysis by the gut lipases can be affected by several factors, including the micronutrient molecular structure [5,26], pH and bile lipid concentration [27,28], and the presence of a minimal amount of dietary fat [29]. Dietary fat stimulates pancreatic juice and biliary secretion, both necessary for lipid digestion and micelle formation, and provide the lipids necessary to structure the mixed micelles. It is assumed that the higher the percentage of lipid micronutrient incorporated in micelles (a percentage called “bioaccessibility”), the higher its absorption efficiency. Although it is assumed that retinol and carotenoids globally transfer to the mixed micelles, some may be incorporated into other proteic or lipid structures (vesicles and liposomes) present in the same aqueous fraction. It has been shown that vitamin A can be incorporated in phospholipid bilayers [30,31] and that vesicle stability to the bile salt deoxycholate is enhanced by the presence of vitamin A [30]. Also, β-lactoglobulin, a lipocalin recovered in cow milk, is able to bind both retinol and β-carotene [32,33,34,35]. It is therefore possible that some proteins found in the diet and/or the pancreatic/biliary secretions bind a fraction of retinol and/or carotenoids and transport them to the brush border membrane (BBM) of the enterocyte. The mechanisms of absorption may then depend on their associated vehicles. In the case of mixed micelles, the particles are isolated from the rest of the intestinal contents in the unstirred water layer of the glycocalyx area and dissociated by pH effect. Indeed, the acidic microclimate of this area promotes the protonation of fatty acids. This phenomenon reduces fatty acid solubility in micelles, causing their release and thus micelle dissociation near the BBM. Components released are then picked up by various more or less specific systems responsible for their uptake by the enterocyte.

Retinol absorption efficiency ranges between 75% [36] and 100% [37,38,39,40,41,42]. Absorption efficiency of β-carotene ranges from 3% to 90% for [43,44,45]. Nevertheless, it is assumed that retinol displays a higher absorption efficiency than carotenoids, as confirmed by recent data obtained in our laboratory using Caco-2 cells. Micellar retinol absorption efficiency was around 30% in 1 h, but less than 5% for micellar provitamin A carotenoids [46]. This may be explained by the presence of an efficient specific transporter for retinol, whereas provitamin A carotenoids are absorbed via non-specific transporters (see below).

## 3. Absorption of Vitamin A and Carotenoids by the Enterocyte (Figure 1)

### 3.1. Apical Uptake and Efflux

Although some authors have suggested that free retinol (from 0.5 to 130 µM) enters intestinal cells by simple diffusion [47], it has long been acknowledged that retinol uptake occurs by a saturable carrier-mediated process at physiological doses, whereas it occurs by passive diffusion at pharmacological doses in Caco-2 cells [48] and in rats [2,4]. A good candidate for retinol specific uptake by intestinal cells was the protein STRA6 (STimulated by Retinoic Acid 6). This 74 kDa multi-transmembrane transporter has been identified as a specific receptor for RBP (Retinol-Binding Protein) [49]. Among other tissues, this protein is found in the intestine during development, although it is not clear whether it persists in adults [50]. Even so, it can still be suggested that STRA6 may be responsible for the uptake of either micellar retinol or retinol bound to protein-like β-lactoglobulin (see Section 1), especially because this protein shares similar amino acid sequence and tertiary structure with RBP. It has also been shown that STRA6 acts as a bidirectional transporter of retinol [51]. Very recently, a new candidate has been discovered: RBPR2 (RBP4-receptor 2). Structurally related to STRA6, this ubiquitous transporter is clearly expressed in the intestine, where it may play a role in dietary retinol uptake [52].

Concerning carotenoids, it has long been assumed that their intestinal absorption occurs by passive diffusion. In living unanesthetized rats, β-carotene absorption was shown to be linear between 0.5 and 11 mM. Also, an increase in the perfusate hydrogen ion concentrations (which should decrease cell membrane resistance to micelle diffusion), additions of different types of fatty acids, or an increase in the perfusate flow rate (which should diminish the thickness of the unstirred water layer) increased its absorption. Conversely, an increase in taurocholate concentration did not change it. These observations led the authors to conclude that β-carotene absorption was driven by passive diffusion [3].

However, a close look at the data obtained by Hollander and coworkers shows a saturation of absorption in the distal part of the intestine. Additionally, the hypothesis of a passive diffusion mechanism for carotenoid uptake cannot explain (i) the high inter-individual variability in absorption observed in human studies [53,54]; (ii) the isomer selectivity and the competition for absorption between carotenoids [55], or between lutein and carotenoids or vitamin, and *vice versa* [56,57] observed in cell models; or (iii) the competition between vitamin E and the carotenoid canthaxanthin described in rats [58]. Finally, the identification of the *Drosophila* gene ninaD encoding a class B scavenger receptor essential for carotenoid cellular distribution [59] also argues in favor of the existence of putative membrane transporters of carotenoids. 

**Figure 1:**
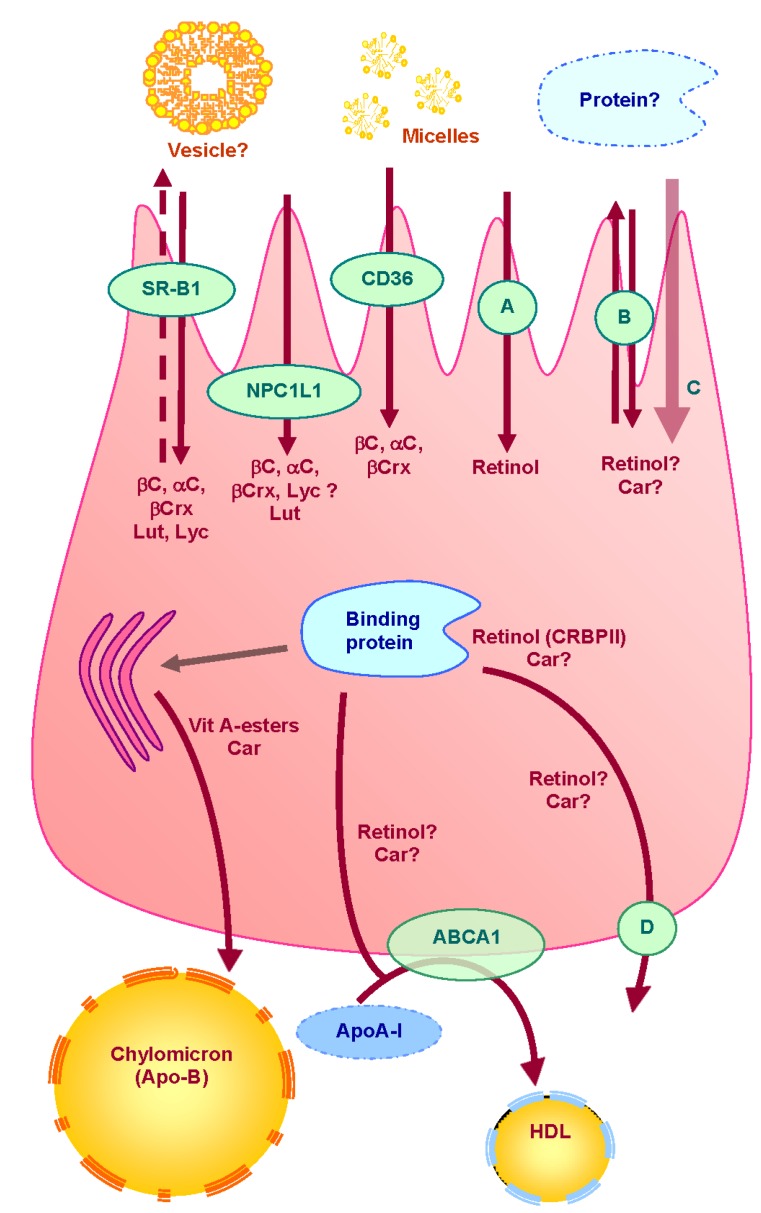
Proteins involved in uptake, transport and secretion pathways of vitamin A and carotenoids across the enterocyte.

Vit = vitamin; βC = β-carotene; αC = α-carotene, βC = β-cryptoxanthine, Lut = lutein; Lyc = lycopene; Car = carotenoids; A = retinol putative specific transporter; B = unidentified apical transporter; C = passive diffusion; D = unidentified basolateral efflux transporter; ? = putative pathway. Carotenoids are captured from mixed micelles by apical membrane transporters: SR-BI, CD36 and NPC1L1. Apical membrane proteins involved in apical uptake of retinol have not yet been identified. A fraction of vitamin A and carotenoids may then be effluxed back to the intestinal lumen via apical membrane transporters (SR-BI and possibly other transporters). Another fraction is transported to the site where they are incorporated into chylomicrons. Some proteins may be involved in intracellular transport of carotenoids, but none has been clearly identified. Conversely, CRBPII has clearly been described as involved in intracellular transport retinol. Retinyl esters and carotenoids are secreted in the lymph into chylomicrons, while a part of the more polar metabolites may be secreted by the portal route. It is suggested that free retinol can also be secreted at the basolateral side via ABCA1 (apoAI pathway).

Several lipid transporters playing a role in carotenoid uptake by the intestinal cell have since been identified. The first one to be highlighted was the Scavenger Receptor class B type I: SR-BI. This ubiquitous 80 kDa single-chain transmembrane glycoprotein is found on the BBM of enterocytes from the duodenum to the colon [60]. First identified as able to bind lipoproteins [61], SR-BI can also facilitate the selective entry into the cell of a large number of ligands, from free and esterified cholesterol to phospholipids and triacylglycerol hydrolysis products [62,63]. However, the effective role of SR-BI in the intestine is probably to facilitate the uptake of lipids other than cholesterol [64]. A first study performed in our laboratory identified this protein as playing a role in the intestinal uptake of the carotenoid lutein in Caco-2 cells [65]. This involvement has since been extended to other carotenoids such as β-carotene [66], zeaxanthin [67] and lycopene [68] in various other tissues. However, SR-BI does not seem to be involved in the uptake of micellar retinol [46].

Another ubiquitous scavenger receptor of interest is CD36 (Cluster Determinant 36). This other 90 kDa single chain-membrane glycoprotein is also expressed at the BBM level of the duodenum and the jejunum [61] and displays a broad substrate specificity [69]. It is assumed to play a key role in fatty acid uptake in the intestine [70]. It was shown that lipid secretion in the lymph was decreased in CD36-deficient mice [71], as CD36 probably allows the routing of the long-chain fatty acids to the endoplasmic reticulum for chylomicron assembly in the enterocyte. Although the underlying mechanism is unknown, it may be linked to the intracellular traffic of the protein between the plasma membrane and the organelles. CD36 was involved in β-carotene uptake using transfected COS cells and mouse BBM vesicles [66], in agreement with the finding that a CD36-related protein is involved in selective carotenoid transport in *Bombyx mori* [72]. In addition, CD36 has been shown to be involved in both lycopene and lutein uptake in mouse 3T3-L1 adipocytes and in mouse adipose tissue cultures [73]. It is noteworthy that CD36 colocalizes with other proteins such as caveolin-1 in lipid rafts [74]. It is therefore possible that a cooperation occurs between these proteins for lipid micronutrient uptake. 

Among the cholesterol membrane transporters, one of the last to be identified was NPC1L1, a 135 kDa protein widely expressed in human tissues including the plasma membrane of the intestinal cell [75,76,77,78,79]. NPC1L1 has been described as the main cholesterol and phytosterol transporter in the intestine [76,77,80]. NPC1L1 was suggested to be involved in carotenoid intestinal uptake, as its specific inhibitor ezetimibe decreased α- and β-carotene uptake by 50%, β-cryptoxanthin and lycopene uptake by 20%, and lutein and zeaxanthin uptake by 7% in Caco-2 cells [81]. Even though this result was confirmed for lutein [82], another study showed that neither ezetimibe nor a blocking antibody raised against NPC1L1 impaired lycopene absorption in the same cells [68].

No clear data are currently available on carotenoid apical efflux across the BBM of the intestinal cell. Nevertheless, as SR-BI can function in both directions in the intestine [83], and as it was shown to be involved in both vitamin D [84] and E [85] efflux throughout Caco-2 cell apical membrane, we suggest that a similar phenomenon can exist for carotenoids. It is also very likely that other transporters, such as the ATP-Binding Cassette (ABC) transporters can act as efflux pumps of lipid micronutrients through the BBM. As a matter of fact, it has been suggested that ABCG5 plays a role in the plasma response to dietary carotenoids [86].

### 3.2. Intracellular Metabolism

Once taken up by the enterocyte, retinol is esterified in retinyl esters by LRAT (Lecithin Retinol Acyl Transferase) and ARAT (Acyl-CoA Acyl Transferase) [87]. The use of LRAT-deficient mice indicated that this enzyme played the most crucial role regarding retinol esterification [88]. The main ester formed is retinyl palmitate, but significant amounts of retinyl oleate, linoleate, and stearate can be found in mice [89] and humans [90].

Interestingly, there is a synergy between STRA6 and LRAT expression [91], although the presence of LRAT is not strictly necessary for retinol influx into the cells [92]. We can hypothesize that such a synergy may also exist with another retinol transporter close to STRA6. If the intestinal cells express both STRA6 or a related transporter and LRAT, then they can theoretically take up more retinol than cells expressing each protein individually, the conversion of retinol into retinyl ester by LRAT within the cell maintaining the driving force for STRA6-mediated retinol uptake.

After uptake by the enterocyte, a substantial quantity of carotenoids is not metabolized (up to 40% of the dietary intake) [93]. A fraction of provitamin A carotenoids is cleaved into retinal by the cytoplasmic protein BCMO1 (β-carotene-15,15′-monooxygenase). Retinal can then be converted to retinol and then to retinyl esters. They can also be cleaved, together with non-provitamin A carotenoids, into apocarotenoids by mitochondrial BCDO2 (β-carotene-9′,10′-dioxygenase) [94]. In order to exhibit a provitamin A activity, a carotenoid should display at least both one β-ionone ring and an appropriate methyl group in its polyene chain. Thus in theory, one molecule of β-carotene can give rise to two molecules of retinol, while α-carotene and β-cryptoxanthine will give one retinol molecule only. In practice, β-carotene is effectively the most potent vitamin A precursor, α-carotene and β-cryptoxanthine showing 30% to 50% of provitamin A activity [95,96]. Apparently, no *cis-trans* isomerization of β-carotene occurs in intestinal cells [55], suggesting that the 9-*cis* isomerization reported *in vivo* [97] occurs in the gastrointestinal lumen.

### 3.3. Cytosolic Transport

Intracellular transport of retinol (and its metabolites retinal and retinoic acid) involve retinoid-binding proteins in most tissues [98,99]. CRBPII (Cellular Retinol-Binding Protein II) is mainly expressed in the absorptive cells of the intestine and is one of the most abundant soluble proteins in the jejunum, representing up to 1% of the total enterocyte cytosolic proteins [100]. It was first shown that its mRNA expression increased in the small intestine of rats under a retinoid-deficient diet [101]. Studies using CRBPII-deficient mice then definitively acknowledged CRBPII as playing an important role in vitamin A intestinal absorption and metabolism [102,103]. The involvement of CRBPI (Cellular Retinol-Binding Protein I) cannot be ruled out, as this protein is also found in the enterocyte [104], but no mechanistic evidence is available to date. STRA6 may be able to couple to both CRBPI and CRBPII depending on the conditions. However, although CRBPII is considered to facilitate the uptake of free retinol by the enterocyte, it has been shown to couple efficiently to STRA6 for retinol efflux [92].

Concerning carotenoids, a cytosolic carotenoid-binding protein (CBP) has been identified in the midgut of the silkworm *Bombyx mori* [72,105]. Interestingly, the lutein-binding protein HR-LBP present in human retina (Human Retinal Lutein-Binding Protein) cross-reacts with antibodies raised against *Bombyx mori* silkworm CBP. If expressed in human enterocytes, LBP would thus be a good candidate as an intracellular transporter of xanthophylls.

Other candidates for the intracellular transport of carotenoids are the apical membrane transporters trafficking between the apical membrane and the cellular organelles. NPC1L1 has been observed in endosomes, perinuclear regions, lysosomes and mitochondria of the human intestinal cell [106,107]. CD36 has been detected in both the apical membrane and the Golgi apparatus [108]. Finally, SR-BI has also been found at the apical and basolateral membranes of enterocytes, as well as in cytoplasmic lipid droplets and in tubulovesicular membranes. Mainly localized in the microvillus membrane in the fasting state, SR-BI seems to be endocytosed after a dietary fat load [109]. Some carotenoids may bind to these apical transporters, or to membrane microdomains close to these transporters, and traffic with them within the cell to be transferred to either other intracellular transporters or to intracellular membranes.

Finally, Fatty Acid-Binding Proteins (FABPs) may be able to participate in the intracellular transport of carotenoids as they display a broad ligand specificity [110,111]. Two FABPs exhibiting a high-affinity binding of long-chain fatty acids are co-expressed in the human enterocyte: intestinal FABP (IFABP) and liver FABP (LFABP) [112,113], and it is suggested that IFABPs allow a specific trafficking of their ligands to their respective metabolic fates. Although dedicated studies are needed to verify whether IFABP and/or LFABP are involved in carotenoid metabolism, it is noteworthy that a genetic association study found that a genetic variant in IFABP was associated with fasting plasma lycopene concentrations [114].

### 3.4. Basolateral Secretion

In the postprandial period, it is assumed that the major fraction of vitamin A and carotenoids are incorporated into chylomicrons that are secreted into the lymph [115,116], vitamin A being recovered as retinyl esters, while carotenoids are recovered in their free forms [90,117]. It was shown in Caco-2 cells that only newly-synthesized retinyl esters could be incorporated into chylomicrons [47], which suggests that retinyl ester synthesis is coupled to chylomicron assembly.

Interestingly, it has been shown that in the fasting state, free retinol unassociated with lipoproteins could be secreted by Caco-2 cells [115]. In addition, in patients who do not assemble and secrete chylomicrons (abetalipoproteinemia), massive retinol supplementation can reverse retinal abnormality due to retinol deficiency [118], indicating that a pathway other than the chylomicron route may be involved in retinol absorption. Additionally, it is now clear that the intestine is also able to secrete large amounts of HDL during the postprandial period via an ABCA1 transporter-dependent pathway [119]. ABCA1 is a 240 kDa protein playing a pivotal role in reverse cholesterol transport, although the molecular mechanisms involved in this phenomenon are still matter of debate [120]. ABCA1 is strongly expressed in the intestine [121], especially at the basolateral side of the cell [122]. Experiments performed in ABCA1-deficient mice showed that it was not significantly involved in the intestinal secretion of retinyl esters [89]. It has been suggested that it could facilitate the efflux of free retinol from intestinal cells [47]. The authors show that both glyburide (an inhibitor of ABC transporters) and SiRNA targeting complementary DNA of ABCA1 partly inhibited retinol basolateral efflux. However, glyburide is far from being a highly specific inhibitor [123], so this result needs further confirmation. 

## 4. Consequences of the Involvement of Vitamin A and Carotenoid Intestinal Transporters

A first factor that may modulate the expression and/or activity of intestinal proteins involved in vitamin A and carotenoid absorption is vitamin A and carotenoids themselves, through a feedback regulation. For example, using both mouse models and human cell lines, it was shown that retinoic acid produced from dietary precursors induced the expression of the intestinal transcription factor ISX (Intestine-Specific Homebox) that repressed the expression of both SR-B1 and BCMO1 [124]. Another example is in Caco-2 cells, where CRBPII expression was increased after retinoic acid treatment [125]. Many transporters can also be regulated by some of their ligands other than retinoids and carotenoids. This is the case for NPC1L1 and the ATP-binding cassette proteins ABCA1, ABCG5 and ABCG8, which are downregulated under cholesterol-free high-fat diets [126]. It was also shown that heart CD36 and hepatic SR-BI expressions were regulated by dietary fat [127,128], and that CRBPII was modulated by diets containing long-chain fatty acids [101]. It is thus possible that the effect of fat on vitamin A and carotenoid absorption described in the first section is actually partly linked to a modulation of the expression of transporters involved in their absorption.

A second factor is the possible existence of genetic variants in genes encoding transport proteins that may affect vitamin A and carotenoid absorption efficiency. Genetic variations leading to modifications in the promoter region of the gene or within the amino acid sequence of the protein may affect its expression and/or activity, and thus its ability to absorb/transport its ligands. This is supported by the broad inter-individual variability observed for carotenoid assimilation [53], and by the associations found between genetic variants SR-BI and CD36 and blood concentrations of carotenoids [129,130]. However, these associations may be due to the effect of genetic variants on the expression or activity of the proteins in tissues other than the intestine (*i.e*., the liver). β-Carotene “low-converter” phenotypes, probably due to genetic variation in the BCMO1 gene, have been reported in several studies [94]. If this hypothesis is supported in the future, it may be worthwhile taking it into account to provide adequate dietary amount of vitamin A and carotenoid to “low responder” or “high responder” phenotypes due to different transport and/or conversion efficacy. It is also interesting to note that as some of the transporters described above are involved in the absorption of several lipid micronutrients (e.g., SR-BI participate in the absorption of carotenoids, but also of vitamin E [85] and vitamin D [84]), some subjects may be at risk of micronutrient multideficiency. Adequate recommended dietary allowances would then be a first step towards personalized nutrition based on the genetic characteristics of individuals.

## 5. Conclusions

To conclude, the full understanding of vitamin A and carotenoid absorption by the enterocyte is still in progress. Although some specific proteins such as the cytosolic CRBPII, and several non-specific transporters such as SR-BI, NPC1-L1, and ABCA1 have been identified, other transporters such as the dietary vitamin A apical membrane transporter remain to be identified. 

## List of abbreviations

ARAT: Acyl-CoA Acyl Transferase
ABCA1: ATP Binding Cassette A1
ABCG5: ATP Binding Cassette G5
ABCG8: ATP Binding Cassette G8
BBM: Brush Border Membrane
BCMO1: β-carotene-15,15′-monooxygenase
BCDO2: β-carotene-9′,10′-dioxygenase
CBP: Carotenoid-Binding protein
CD36: Cluster Determinant 36
CRBPII: Cellular Retinol Binding Protein II
HR-LBP: Human Retinal Lutein-Binding Protein
ISX: Intestine-Specific Homebox
L-FABP: Liver Fatty-Acid-Binding Protein
LRA: Lecithin Retinol Acyl Transferase
NPC1L1: Niemann-Pick C1-Like 1
RBP: Retinol Binding Protein
RBPR2: RBP-receptor 2
SR-BI: Scavenger Receptor class B type 1
STRA6: STimulated by Retinoic Acid 6
